# The association of polymorphisms in lncRNA-*H19* with hepatocellular cancer risk and prognosis

**DOI:** 10.1042/BSR20171652

**Published:** 2018-09-05

**Authors:** Ming-li Yang, Zhe Huang, Qian Wang, Huan-huan Chen, Sai-nan Ma, Rong Wu, Wei-song Cai

**Affiliations:** 1 The Second Oncology Department of Affiliated Shengjing Hospital of China Medical University, Shenyang 110022, China; 2 Genery Surgery Department of Affiliated Shengjing Hospital of China Medical University, Shenyang 110015, China

**Keywords:** hepatocellular cancer, prognosis, risk, Single nucleotide polymorphism

## Abstract

Hepatocellular cancer (HCC) is one of the major causes of cancer-related mortality. Genetic polymorphisms may affect the susceptibility and clinical outcomes of cancers. We aim to manifest the association of single nucleotide polymorphisms (SNPs) of lncRNA-*H19* gene with the risk and prognosis of HCC. A total of 944 samples composed of 472 HCC patients and 472 matched controls were included in the risk analysis and amongst them 350 HCC samples were investigated in the prognosis analysis. KASP method was conducted for the SNP genotyping. The TT + CT genotype of rs2839698 was found to be associated with a 1.32-fold increased HCC risk (*P*=0.037, 95% confidence interval (CI) = 1.02–1.70). In the stratified analysis, rs2839698 (odds ratio (OR) = 1.57, *P*=0.007, 95% CI = 1.13–2.18) and rs3024270 (OR = 1.71, *P*=0.019, 95% CI = 1.09–2.68) were found to show more obvious increased HCC risk in the age ≤60 subgroup. And we found that rs2839698 showed an increased HCC risk in the ever smoking subgroup. But in the male subgroup of rs2735971, it showed a decreased HCC risk. Furthermore, haplotype analysis showed that rs2735971-rs2839698-rs3024270 G-T-C significantly increased the risk of HCC (OR = 1.23, 95% CI = 1.01–1.51, *P*=0.043). Multilogistic analysis revealed no significant results of the interaction effects of the SNPs and environment factors. And in our study, rs2839698 showed a significant poor prognosis in the ever smoking subgroup (hazard rate (HR) = 5.19, 95% CI = 1.12–24.07, *P*=0.035). lncRNA-*H19* rs2839698 SNP has the potential to be predictors for HCC risk and prognosis.

## Introduction

Hepatocellular cancer (HCC) is the major liver malignancy that attributes toward the second foremost cause of cancer-related mortality worldwide [1]. Individual hereditary and environmental factors proved to be associated with the incidence of HCC [2]. So far, there are many single nucleotide polymorphisms (SNPs) that have been reported to be related to HCC risk in some coding and non-coding genes and have manifested great significance for the selection of individuals who would benefit from the specific diagnostic and preventative measures [3]. However, few studies investigated the role of lncRNA polymorphisms as a precaution biomarker for HCC risk and prognosis. Furthermore, many studies have reported that the gene polymorphisms could serve as the predictor of the diagnosis and prognosis of cancers [4,5], suggesting a valuable application for the diagnosis and prognosis associated with polymorphisms.

In human genome, there are approximately 5–10% sequences transcribed constantly, and only approximately 1% are protein-coding sequences while a large part of others are non-coding RNAs (ncRNAs) [6]. lncRNA, larger than 200 nts, is one of the most important members of the ncRNA family and has been identified as abnormally altered in the genes and differently expressed in tumors [7,8]. The lncRNA-*H19*, located on chromosome 11p15.5 [9], was reported to be one of the major genes in cancer [10]. Many studies have reported that *H19* as an oncogene lncRNA in multiple cancers, such as, colorectal cancer [11], gastric cancer [12], breast cancer [13], bladder cancer [14], and so on. In addition, recent researches have proved that lncRNA-*H19* plays important role in cancer initiation, progression, metastasis, and indicates poor prognosis and promotes tumor growth [11,15,16].

It is well accepted that lncRNA-*H19* works importantly in the incidence and prognosis of cancers and SNPs in *H19* can be used as a promising biomarker for cancers risk [17]. Recently, a meta-analysis for the association of *H19* polymorphisms and cancer risk had published [18], but the interaction of *H19* SNPs and environmental factors as well as the association of *H19* SNPs and the cancer prognosis were not analyzed further. And there is still no investigation about the *H19* polymorphism associated with both HCC risk and prognosis. Whether lncRNA-*H19* polymorphisms play some roles in HCC and could be promising biomarkers for the risk and prognosis of HCC, it is still not clear.

In the present study, we selected three potential functional SNPs in lncRNA-*H19* gene according to the candidate gene association study strategy to explore the relationship between *H19* polymorphism and HCC risk and prognosis. We aimed to manifest predictive biomarkers for risk and prognosis of HCC and provide the basic for the use of *H19* gene polymorphisms as precautionary biomarkers of individuals and improve the comprehension of the etiology and disease development of HCC.

## Materials and methods

### Patients and study design

This research project was approved by the Ethical Committee of the Shengjing Hospital of the China Medical University and written informed consent was obtained. The present study was designed as two independent but related parts including risk research and prognosis research. In the risk study, a total of 944 participants were involved, including 472 HCC patients and 472 sex and age (±5) frequency-matched controls from the Shengjing Hospital of China Medical University from 2013 to 2015. The response rate for cases and controls are up to 90% or more.

For the aim to manifest the relationship between lncRNA-*H19* polymorphisms and overall survival in HCC patients, we conducted the research with the data of 350 HCC patients, whose information of death and survival was available for analysis. The HCC patients had pathologically confirmed HCC. Patients (i) with distant metastasis found preoperatively, (ii) who underwent preoperative radiotherapy or chemotherapy, or (iii) with incomplete pathological data entries were excluded from the prognosis analysis. Follow-up was completed by 10 July, 2017.

### Polymorphisms’ sites selected

The studied polymorphisms of lncRNA-*H19* were selected by the HapMap data [19]. TagSNPs were selected by Tagger via Haploview with the following criteria: pairwise tagging of HapMap population with r^2^ ≥ 0.8; a minor allele frequency (MAF) ≥5%; and Chinese Han Beijing (CHB) ethnicity. And we expanded 10 kbp both upstream and downstream of *H19*. Then, 17 SNPs were included as candidate SNPs (Supplementary Figure S1 and Materials), and we referred a published literature [17] and took the intersection as the considering promising aiming SNPs. Ultimately, there were three SNPs covering lncRNA-H19 gene selected to proceed our study which were rs2735971 (G→A), rs2839698 (C→T), rs3024270 (G→C).

### Genotyping

Genomic DNA was extracted by the method of literature [20] and was diluted to working concentrations of 20 ng.μl^−1^ for genotyping. The genotyping assay was performed by Gene Company using KASP (Gene Company, Shanghai, China). The information of KASP primers was summarized in the Supplementary Table S1. Five percent of the whole samples were repeatedly genotyped, the concordance rate of the repeated cases performed 100% which suggested that the genotyping results were reliable.

### Statistical analysis

χ^2^ test was used to compare the demographic characteristics of samples and ANOVA was conducted for age variability. Multivariate logistic regression with adjustments for age and gender was proceeded to calculate the association of the selected SNPs and HCC risk. SHEsis software was used to analyze the haplotype of the selected gene [21]. The analysis of polymorphisms and clinical parameters was performed by χ^2^ test. Univariate and multivariate survival analysis was conducted by the log-rank test and the Cox proportional hazards model. Statistical analysis was performed by using SPSS version 18.0 software (SPSS, Chicago, IL, U.S.A.) and *P*-value <0.05 was considered to be significantly statistical.

## Results

### The association of lncRNA-H19 SNPs with HCC risk

The demographic characteristics of HCC and controls are shown in Supplementary Table S2. In Table 1, it showed all the polymorphisms genotype distributions of both cases and controls, including three lncRNA-*H19* SNPs (rs2735971, rs2839698, rs3024270) which were all conformed to Hardy–Weinberg equilibrium (HWE).

**Table 1:** The association of *lncRNA* gene SNPs and risk of HCC

Gene	Chr. pos.	SNP^a^	Loc.	Genotype	Controls (%)	Cases (%)	*P*^b^	OR (95% CI)	*P*_HWE_
**Recognition-related**									
*H19*	11p15.5	rs2735971	Intron	GG	313 (67.31)	327 (70.32)		1 (Ref.)	0.697
				AG	139 (29.89)	126 (27.10)	0.336	0.87 (0.65–1.16)	
				AA	13 (2.80)	12 (2.58)	0.824	0.91 (0.41–2.04)	
		rs2839698	Intron	CC	245 (53.03)	215 (46.14)		1 (Ref.)	0.297
				CT	185 (40.04)	211 (45.28)	0.058	1.30 (0.99–1.70)	
				TT	32 (6.93)	40 (8.58)	0.157	1.44 (0.87–2.37)	
				TT + CT *vs* CC			**0.037**	1.32 (1.02–1.70)	
				T *vs* C			**0.044**	1.23 (1.01–1.50)	
		rs3024270	Intron	GG	170 (36.48)	151 (32.06)		1 (Ref.)	0.247
				CG	215 (46.14)	225 (47.78)	0.254	1.18 (0.89–1.58)	
				CC	81 (17.38)	95 (20.16)	0.141	1.32 (0.91–1.91)	

Abbreviations: Chr. Pos., chromosomal position; CI, confidence interval; Loc., localization; OR, odds ratio; *P*_HWE_, *P*-value for HWE.

^a^The sort order was according to the SNP location in its genes from 5′ to 3′ ends.

^b^*P*-value was calculated by adjusting age and gender. The bold text in this table means the *P*<0.05 and is significant.

LncRNA-*H19* rs2839698 polymorphism was calculated to be associated with an increased risk of HCC. In the dominant model, rs2839698 TT + TC genotype appeared with a 1.32-fold increased HCC risk when compared with CC genotype (*P*=0.037, Table 1). Subsequently, we conducted stratified analysis by the factors of gender, age, smoking, and drinking to manifest the relationships between every SNP and HCC risk. The results displayed in Table 2 indicated the potential predicting values for specific subgroup populations. When stratified by gender, rs275971 showed a decreased HCC risk tendency in AG genotype of male subgroup for odds ratio (OR) = 0.72, *P*=0.048. In the subgroup stratified by age, rs2839698 showed an obvious HCC risk tendency in CT genotype of age ≤60 subgroup (OR = 1.57, *P*=0.007) and the similar situation was showed in rs3024270 CC genotype of age ≤60 subgroup (OR = 1.71, *P*=0.019). When stratified by smoking factor, rs2839698 showed a more obvious HCC tendency in CT subgroup of ever smoker subgroup (OR = 1.89, *P*=0.041, Table 2).

**Table 2:** The association of lncRNA polymorphisms and hepatocellular risk stratified by host characteristics

Variables	Genotype	HCC compared with CON	*P*	OR (95% CI)
H19 rs2735971				
Gender				
Male	GG	276/248		1 (Ref.)
	AG	93/117	**0.048**	**0.72 (0.52–1.00)**
	AA	9/9	0.791	0.88 (0.34–2.26)
Female	GG	51/65		1 (Ref.)
	AG	33/22	0.058	1.92 (0.98–3.78)
	AA	3/4	0.804	0.82 (0.17–4.05)
Age				
≤60	GG	205/230		1 (Ref.)
	AG	87/102	0.811	0.96 (0.68–1.35)
	AA	8/7	0.537	1.35 (0.48–3.81)
>60	GG	122/83		1 (Ref.)
	AG	39/37	0.160	0.68 (0.40–1.17)
	AA	4/6	0.216	0.44 (0.12–1.62)
Smoking				
Ever smoker	GG	56/80		1 (Ref.)
	AG	19/43	0.105	0.58 (0.30–1.12)
	AA	3/4	0.952	1.05 (0.23–4.89)
Never smoked	GG	152/128		1 (Ref.)
	AG	62/62	0.357	0.81 (0.53–1.26)
	AA	7/6	0.886	1.09 (0.33–3.63)
Alcohol drinking				
Drinker	GG	32/57		1 (Ref.)
	AG	11/33	0.171	0.57 (0.25–1.28)
	AA	3/1	0.157	5.39 (0.52–55.55)
Non-drinker	GG	175/151		1 (Ref.)
	AG	70/71	0.413	0.84 (0.56–1.27)
	AA	7/9	0.485	0.68 (0.24–1.98)
H19 rs2839698				
Gender				
Male	CC	176/195		1 (Ref.)
	CT	168/152	0.191	1.22 (0.91–1.65)
	TT	33/24	0.147	1.52 (0.86–2.68)
Female	CC	39/50		1 (Ref.)
	CT	43/33	0.092	1.74 (0.91–3.30)
	TT	7/8	0.729	1.23 (0.39–3.91)
Age				
≤60	CC	135/185		1 (Ref.)
	CT	143/125	**0.007**	**1.57 (1.13–2.18)**
	TT	25/25	0.276	1.40 (0.78–2.55)
>60	CC	80/60		1 (Ref.)
	CT	68/60	0.528	0.86 (0.53–1.39)
	TT	15/7	0.320	1.63 (0.62–4.27)
Smoking				
Ever smoker	CC	29/65		1 (Ref.)
	CT	40/50	**0.041**	**1.89 (1.03–3.48)**
	TT	9/10	0.155	2.08(0.76–5.68)
Never smoked	CC	112/105		1 (Ref.)
	CT	88/78	0.917	1.02 (0.67–1.55)
	TT	22/12	0.121	1.88 (0.85–4.16)
Alcohol drinking				
Drinker	CC	19/45		1 (Ref.)
	CT	22/40	0.402	1.39 (0.65–2.97)
	TT	5/4	0.088	3.68 (0.82–16.46)
Non-drinker	CC	121/124		1 (Ref.)
	CT	106/88	0.360	1.20 (0.81–1.76)
	TT	26/18	0.166	1.61 (0.82–3.15)
H19 rs3024270				
Gender				
Male	GG	129/136		1 (Ref.)
	CG	181/173	0.573	1.10 (0.80–1.51)
	CC	71/64	0.452	1.17 (0.77–1.78)
Female	GG	22/34		1 (Ref.)
	CG	44/42	0.092	1.86 (0.90–3.82)
	CC	24/17	0.051	2.39 (1.00–5.73)
Age				
≤60	GG	93/128		1 (Ref.)
	CG	145/157	0.153	1.40 (0.88–2.21)
	CC	67/56	**0.019**	**1.71 (1.09–2.68)**
>60	GG	58/42		1 (Ref.)
	CG	80/58	0.151	1.29 (0.91–1.84)
	CC	28/25	0.438	0.76 (0.39–1.51)
Smoking				
Ever smoker	GG	17/44		1 (Ref.)
	CG	44/57	0.055	1.96 (0.99–3.89)
	CC	18/24	0.122	1.93 (0.84–4.42)
Never smoked	GG	74/69		1 (Ref.)
	CG	100/94	0.802	0.94 (0.60–1.48)
	CC	50/36	0.310	1.34 (0.76–2.34)
Alcohol drinking				
Drinker	GG	13/26		1 (Ref.)
	CG	23/48	0.921	0.96 (0.41–2.22)
	CC	10/16	0.553	1.37 (0.48–3.93)
Non-drinker	GG	77/87		1 (Ref.)
	CG	121/102	0.186	1.33 (0.87–2.01)
	CC	58/44	0.099	1.53 (0.92–2.55)

Abbreviation: CI, confidence interval. The bold text means the significant results.

We further analyzed the relationship between lncRNA-*H19* SNPs haplotype and HCC risk and found that rs2735971-rs2839698- rs3024270 G-T-C significantly increased the risk of HCC (OR = 1.23, confidence interval (CI) = 1.01–1.51, *P*=0.043) (Table 3).

**Table 3:** The association of haplotype of *lncRNA* gene and HCC risk

Haplotype	Control (%)	Case (%)	*P*	OR (95% CI)
H19^a^				
ACC*	100.87 (0.110)	110,67 (0.122)	0.461	0.90 (0.67–1.20)
ACG*	47.08 (0.051)	49.23 (0.054)	0.812	0.95 (0.63–1.44)
GCG*	460.01 (0.501)	485.37 (0.537)	0.207	0.89 (0.74–1.07)
GTC*	282.04 (0.307)	242.50 (0.268)	**0.043**	**1.23 (1.01–1.51)**

Haplotype for ^a^, H19 rs2735971-rs2839698-rs3024270. The bold text means the significant results.

### LncRNA-H19 SNP–environment interaction with HCC risk

Data mining was conducted to analysis the possible association between interaction model for lncRNA-*H19* polymorphisms and environmental factors in HCC risk (Table 4) and found that there were no significant results.

**Table 4:** The interaction effect of H19 SNPs and environmental factors

Gene	Location	SNP	*P*_interaction_ with smoking	*P*_interaction_ with drinking
*H19*	11p15.5	rs2735971	0.535	0.775
		rs2839698	0.190	0.755
		rs3024270	0.822	0.973

### The association of lncRNA-H19 SNPs with HCC prognosis

The association of HCC patient clinical features and univariate analysis of overall survival was shown in Supplementary Table S3. We also analyzed the relationship of each lncRNA-*H19* SNPs and the overall survival of HCC, there existed no significant association between the SNPs and the survival of HCC either in the univariate or multivariate survival analysis (Table 5). In the stratified analysis, we found that the rs2839698 showed a significant poor prognosis in the ever smoking subgroup (hazard rate (HR) = 5.19, CI = 1.12–24.07, *P*=0.035, Table 6).

**Table 5:** Univariate and multivariate cox proportional hazard analysis for H19 SNPs on HCC prognosis

Variables			All HCC, *n* (%)	Deaths, *n*	MST^a^ (M)	Univariate *P*-value	Hazard ratio (95% CI)	Multivariate^c^ *P*-value	Hazard ratio (95% CI)
			*n*=286	*n*=156					
H19	rs2735971	GG	193 (67.48)	117 (75)	47.000		1 (Ref.)		1 (Ref.)
		AG	85 (29.72)	37 (23.72)	52.000	0.769	1.06 (0.72–1.57)	0.752	0.94 (0.64–1.39)
		AA	8 (2.80)	2 (1.28)	33.000	0.756	1.12 (0.55–2.26)	0.763	0.80 (0.20–3.30)
			*n*=344	*n*=136					
	rs2839698	CC	182 (52.9)	69 (50.7)	56.000		1 (Ref.)		1 (Ref.)
		CT	144 (41.9)	61 (44.9)	48.000	0.776	0.95 (0.67–1.34)	0.716	1.07 (0.75–1.51)
		TT	18 (5.2)	6 (4.4)	78.4^b^	0.501	1.16 (0.76–1.76)	0.499	0.75 (0.32–1.73)
			*n*=349	*n*=137					
	rs3024270	GG	59 (16.9)	21 (15.3)	90.000		1 (Ref.)		1 (Ref.)
		CG	159 (45.6)	67 (48.9)	48.000	0.716	0.91 (0.56–1.49)	0.887	1.03 (0.71–1.49)
		CC	131 (37.5)	49 (35.8)	56.000	0.831	0.97 (0.75–1.26)	0.808	0.94 (0.56–1.58)

^a^, MST, median survival time (months).

^b^, Mean survival time was provided when MST could not be calculated.

^c^, Multivariate survival analysis was carried out by adding the age and gender variable to the clinicopathological parameters with *P*<0.05.

**Table 6:** Univariate proportional hazard analysis stratified by host characteristics for the association of H19 polymorphisms and HCC

Gene	SNP	Stratified	Stratified factors	Genotype	HCC (*n* (%))	Deaths (*n*)	MST^a^(M)	*P*-value	Hazard ratio (95% CI)
H19	rs2735971								
		Gender	Male	GG	160 (69.57)	67	47.0		1 (Ref.)
				AG	64 (27.83)	28	52.0	0.669	0.91 (0.58–1.41)
				AA	6 (2.60)	2	33.0	0.778	1.23 (0.30–5.04)
			Female	GG	33 (58.93)	13	38.0		1 (Ref.)
				AG	21(37.50)	9	32.0	0.918	1.05 (0.45–2.45)
				AA	2 (3.57)	0	NA	NA	NA
		Age	≤60	GG	109 (63.01)	43	47.0		1 (Ref.)
				AG	58 (33.53)	28	48.0	0.599	1.14 (0.71–1.83)
				AA	6 (3.46)	1	25.8^b^	0.844	0.82 (0.11–6.03)
			>60	GG	84 (74.34)	37	45.0		1 (Ref.)
				AG	27 (23.89)	9	56.0	0.276	0.67 (0.32–1.38)
				AA	2 (1.77)	1	33.0	0.847	0.82 (0.11–6.02)
		Smoking	Ever smoker	GG	17 (62.96)	6	97.1^b^		1 (Ref.)
				AG	8 (29.63)	1	117.2^b^	0.264	0.30 (0.04–2.49)
				AA	2 (7.41)	0	NA	NA	NA
			Never smoked	GG	99 (66.44)	35	81.3^b^		1 (Ref.)
				AG	46 (30.87)	20	32.0	0.702	1.11 (0.64–1.93)
				AA	4 (2.69)	1	33.0	0.665	0.65 (0.09–4.72)
		Alcohol drinking	Drinker	GG	9 (60.00)	2	NA		1 (Ref.)
				AG	4 (26.67)	0	NA	NA	NA
				AA	2 (13.33)	0	NA	NA	NA
			Non-drinker	GG	107 (66.46)	39	84.5^b^		1 (Ref.)
				AG	50 (31.06)	21	48.0	0.919	0.97 (0.57–1.66)
				AA	4 (2.48)	1	33.0	0.648	0.63 (0.09–4.59)
		HBV	Positive	GG	86 (65.15)	31	88.1^b^		1 (Ref.)
				AG	40 (30.30)	13	90.0	0.331	0.73 (0.38–1.39)
				AA	6 (4.55)	1	64.1^b^	0.552	0.55 (0.07–4.02)
			Negative	GG	19 (63.33)	7	27.0		1 (Ref.)
				AG	11 (36.67)	4	25.7^b^	0.803	1.17 (0.34–4.09)
				AA	0	0	NA	NA	NA
H19	rs2839698								
		Gender	Male	CC	157 (56.07)	61	56.0		1 (Ref.)
				CT	110 (39.29)	50	47.0	0.692	1.08 (0.74–1.57)
				TT	13 (4.64)	2	97.5^b^	0.087	0.29 (0.07–1.20)
			Female	CC	25 (39.06)	8	55.0		1 (Ref.)
				CT	34 (53.13)	11	51.0	0.909	1.05 (0.42–2.63)
				TT	5 (7.81)	4	5.0	0.070	3.14 (0.91–10.85)
		Age	≤60	CC	111 (51.63)	42	56.0		1 (Ref.)
				CT	94 (43.72)	43	48.0	0.614	1.12 (0.73–1.71)
				TT	10 (4.65)	2	91.4^b^	0.233	0.42 (0.10–1.75)
			>60	CC	71 (55.04)	27	56.0		1 (Ref.)
				CT	50 (38.76)	18	79.4^b^	0.838	0.94 (0.52–1.71)
				TT	8 (6.20)	4	27.0	0.681	1.25 (0.44–3.58)
		Smoking	Ever smoker	CC	16 (50.00)	2	127.1^b^		1 (Ref.)
				CT	16 (50.00)	9	45.0	**0.035**	**5.19 (1.12–24.07)**
				TT	0	0	NA	NA	NA
			Never smoked	CC	97 (57.06)	31	69.0		1 (Ref.)
				CT	59 (34.71)	23	47.0	0.624	1.15 (0.67–1.97)
				TT	14 (8.23)	5	75.1^b^	0.792	0.88 (0.34–2.28)
		Alcohol drinking	Drinker	CC	12 (63.16)	1	70.2^b^		1 (Ref.)
				CT	7 (36.84)	4	45.0	0.110	5.99 (0.67–53.96)
				TT	0	0	NA	NA	NA
			Non-drinker	CC	101 (55.19)	32	69.0		1 (Ref.)
				CT	68 (37.16)	28	48.0	0.339	1.28 (0.77–2.13)
				TT	14 (7.65)	5	75.1^b^	0.927	0.96 (0.37–2.47)
		HBV	Positive	CC	70 (52.24)	19	94.3^b^		1 (Ref.)
				CT	55 (41.04)	24	52.0	0.125	1.60 (0.88–2.93)
				TT	9 (6.72)	3	78.7^b^	0.924	0.94 (0.28–3.20)
			Negative	CC	17 (56.67)	6	42.2^b^		1 (Ref.)
				CT	11 (36.67)	3	58.3^b^	0.882	0.90 (0.22–3.65)
				TT	2 (6.66)	2	2.0	0.229	2.74 (0.53–14.18)
H19	rs3024270								
		Gender	Male	GG	114 (40.14)	45	56.0		1 (Ref.)
				CG	131 (46.13)	57	48.0	0.946	0.99 (0.67–1.46)
				CC	39 (13.73)	12	90.0	0.233	0.68 (0.36–1.28)
			Female	GG	17 (26.15)	4	55.0		1 (Ref.)
				CG	28 (43.08)	10	51.0	0.532	1.45 (0.45–4.65)
				CC	20 (30.77)	9	27.0	0.086	2.83 (0.87–9.28)
		Age	≤60	GG	79 (36.41)	28	86.7^b^		1 (Ref.)
				CG	99 (45.62)	44	48.0	0.926	1.02 (0.64–1.65)
				CC	39 (17.97)	15	90.0	0.611	1.18 (0.63-2.21)
			>60	GG	52 (39.39)	21	55.0		1 (Ref.)
				CG	60 (45.46)	23	76.8^b^	0.946	0.98 (0.54–1.77)
				CC	20 (15.15	6	56.6^b^	0.370	0.66 (0.27–1.64)
		Smoking	Ever smoker	GG	7 (21.21)	0	NA		1 (Ref.)
				CG	21 (63.64)	10	NA	NA	NA
				CC	5 (15.15)	1	NA	NA	NA
			Never smoked	GG	65 (37.79)	20	86.3^b^		1 (Ref.)
				CG	71 (41.28)	27	47.0	0.710	1.12 (0.63–1.99)
				CC	36 (20.93)	13	90.0	0.978	1.01 (0.50–2.04)
		Alcohol drinking	Drinker	GG	7 (36.84)	0	NA		1 (Ref.)
				CG	10 (52.63)	5	NA	0.298	NA
				CC	2 (10.53)	0	NA	NA	NA
			Non-drinker	GG	65 (34.95)	20	91.3^b^		1 (Ref.)
				CG	82 (44.09)	32	69.0	0.518	1.20 (0.69–2.11)
				CC	39 (20.96)	14	90.0	0.939	1.03 (0.52–2.04)
		HBV	Positive	GG	45 (32.61)	13	95.0^b^		1 (Ref.)
				CG	63 (45.65)	25	69.0	0.398	1.34 (0.68–2.62)
				CC	30 (21.74)	9	90.0	0.951	0.97 (0.42–2.28)
			Negative	GG	12 (40.00)	4	44.7^b^		1 (Ref.)
				CG	13 (43.33)	4	51.5^b^	0.786	1.22 (0.29–5.05)
				CC	5 (16.67)	3	27.0	0.358	2.03 (0.45–9.20)

NA, not available. The bold text means the significant results. ^a^, MST, median survival time (months); ^b^, Mean survival time was provided when MST could not be calculated.

## Discussion

*H19* gene, with the length of 2.3 kb and located in 11p15.5, containing five exons and three introns [22], which has been well accepted that lncRNA-*H19* plays an important role in the development, migration, invasion, and metastasis of cancers [23]. As a long ncRNA, *H19* lacks the ORF to translate protein, however, the end product of which is RNA sequence and can also participate in RNA regulation [24]. Due to the relationship between *H19* variants and cancer risk as well as prognosis is still needed to be clarified; the *H19* polymorphisms have been of great interest in the recent years [17]. Many studies have been reported that *H19* SNPs were related to cancers risk and prognosis, such as, rs217727 with breast cancer [13], rs2389698 with gastric cancer [25], but rs1859168 was reported to reduce the risk of pancreatic cancer [26]. However, the association between lncRNA-*H19* SNPs and HCC risk and prognosis is still unreported.

In order to research the role of *H19* SNPs in HCC risk and prognosis, we screened three intron SNPs in *H19* gene. Under the dominant model, TT + CT genotype of rs2389698 was found to be 1.32-fold increased HCC risk compared with CC wild-type; this is the first report indicating that *H19* SNPs was related to HCC risk. Thus, rs2389698 may serve as a promising predictor for HCC risk. The rs2389698 was an intron SNP and it is accepted now that intron SNP also had its possibly own functions such as affecting selective splicing [27,28]. Because some intron polymorphisms had some important location and even function, it is believable that choosing intron SNP for research is also a good choice such as this rs2389698 SNP.

When stratified by gender, age, smoking, and drinking factors, a more obvious OR of 1.57 and 1.71 was shown for the rs2389698 and rs3024270 in the age ≤60 subgroup, respectively. And in the ever-smoking subgroup of rs2389698, it showed 1.89-fold increased HCC risk. It was reported that the expression of *H19* could be induced by cigarette smoke condensate in human respiratory epithelial cells [29]. Thus, we suppose that the mature lncRNA-H19 could be affected by cigarette smoke and when in the ever-smoking subgroup, the polymorphisms could contribute to more functions than the environmental factors. These results indicated that the promising SNPs of *H19* may be better biomarkers for the certain subgroup and could bring benefit to the individualized diagnosis for HCC in the certain population.

Furthermore, we performed interaction analysis for the multiple lncRNA-*H19* SNPs and environmental factors including smoking and drinking. Yet there showed no interaction between the three polymorphisms and environmental factors. And in the lncRNA-*H19* SNPs haplotype and HCC risk analysis, rs2735971-rs2839698-rs3024270 G-T-C were found significantly increased the risk of HCC (OR = 1.23, CI = 1.01–1.51, *P*=0.043). These results suggested that the G-T-C haplotype suffers more risk than other haplotype.

We further performed univariate and multivariate Cox proportional hazards regression analysis of overall survival time to explore the association between lncRNA-*H19* SNPs and HCC prognosis. No significant association was found between *H19* SNPs and HCC overall survival. In addition, we performed subgroup analysis for HCC prognosis and found rs2839698 that showed significant poor survival condition in the ever-smoking subgroup which suggested that it may affect HCC prognosis and could be a promising biomarker for HCC prognosis. As discussed above, the expression of *H19* could be induced by cigarette smoke [29]. Thus, when in the ever-smoking subgroup, the polymorphisms could contribute more functions than the environmental factors and individuals carrying the variant genotype which also had a higher incidence of cancer risk could have a poorer survival of HCC.

However, there still existed several limitations in the present study. First, the sample size was relatively not large enough for the analysis which may limit the possible analysis of other subgroup analysis and interaction analysis for variant genotype. Second, we only studied the local population and did not include residents of other areas. Third, because the controls were collected from the health check program of our hospital and there was no message of the HBV for them which could not assess the influence of this factor. In future, larger sample and multicenter samples are needed for the confirmation study of our findings.

In conclusion, we found an intron rs2839698 SNP of lncRNA-*H19* was associated with an increased risk of HCC. And more significant findings were shown in the age ≤60 subgroup in rs2839698 and rs3024270. In the ever-smoking subgroup, rs2839698 showed an obvious increased HCC risk too. But we got an adverse result in the male subgroup of rs2735971 SNP that showed a decreased HCC risk. In addition, we found that the rs2735971-rs2839698- rs3024270 G-T-C significantly increased the risk of HCC in the analysis of haplotype and HCC risk. And the MDR analysis had no significant findings. In the prognosis analysis, the rs2839698 showed a poor prognosis in the ever-smoking subgroup. In the future, the larger scale sample experiments and analyses are needed to confirm our results.

## Supporting information

**Supplementary Figure S1:** The LD figure of the H19 genetic polymorphisms

**Supplementary Table S1.** The primier information for the H19 polymorphisms

**Supplementary Table S2.** The baseline of the subjects

**Supplementary Table S3.** HCC patient clinical features and univariate analysis of overall survival

## Abbreviations

CI: confidence interval
HCC: hepatocellular cancer
HR: hazard rate
ncRNA: non-coding RNA
OR: odds ratio
SNP: single nucleotide polymorphism
lncRNA-H19: Long non-coding RNA-H19
KASP: kompetitive allele specific polymerase chain reaction
MDR: multifactor dimensionality reduction
