# Supplementary material for: The association of polymorphisms in lncRNA-H19 with hepatocellular cancer risk and prognosis
Source: Biosci Rep. 2018 Sep 5;38(5):BSR20171652. doi: 10.1042/BSR20171652 (PMC6123070; doi:10.1042/BSR20171652)
Supplement: Supplementary file 1 [file bsr20171652_Supp1.pdf]

The Association of Polymorphisms in lncRNA-*H19* with Hepatocellular Cancer Risk and Prognosis

Ming-li Yang[1], Zhe Huang[2], Qian Wang[1], Huan-huan Chen[1], Sai-nan Ma[1], Rong Wu[1], Wei-song Cai [1,*]

[1] The 2nd Oncology Department of Affiliated Shengjing Hospital of China Medical University, Shenyang 110022, China

[2]Genery Surgery Department of Affiliated Shengjing Hospital of China Medical University, Shenyang 110015, China

***Corresponding author:** Dr. Wei-song Cai, the 2nd Oncology Department of Affiliated Shengjing Hospital of China Medical University, Huaxiang Road 39#, Tiexi District, Shenyang 110022, China. Telephone：+86-024-96615-63215. Email：cailab9@hotmail.com.

The contents:

Supplementary Table S1. The primier information for the *H19* polymorphisms

Supplementary Table S2, The baseline of the subjects

Supplementary Table S3, HCC patient clinical features and univariate analysis of overall survival

Supplementary Figure S1. The LD figure of the *H19* genetic polymorphisms

Supplementary Material. The results of the haploview software for the selection of the *H19* genetic polymorphisms

Supplementary Table S1. The primier information for the H19 polymorphisms

ID	Primer_AlleleFAM	Primer_AlleleHEX	Primer_Common	AlleleFAM	AlleleHEX
rs2735971	CCGATTCCACAACTACAACCAATTCT	CGATTCCACAACTACAACCAATTCC	CTTCAGTCTCACCGCCCGGAT	A	G
rs2839698	AGTGAGGAGTGTGGAGTAGGC	CAGTGAGGAGTGTGGAGTAGGT	GATGTCGCCCTGTCTGCACGAT	C	T
rs3024270	CCACCATCTCACTGCCCCG	CCACCATCTCACTGCCCCC	CACCAGCCTAAGGTGTTCTGTAGAA	G	C

Supplementary Table S2, The baseline of the subjects

Variables	Hepatocellular cancer patients (%)	Controls (%)	*P*
	n=472	n=472	
Gender			0.805
Male	382(80.9)	379(80.3)	
Female	90(17.3)	93(19.7)	
Age			0.821
Mean±SD	56.6±10.8	56.7±7.2	
Median	57	56	
Range	21-90	26-85	
Smoking			**<0.001**
Never	224(73.9)	199(60.7)	
Ever	79(26.1)	129(39.3)	
Drinking			**<0.001**
Never	256(84.8)	235(71.9)	
Ever	46(15.2)	92(28.1)	
HBsAg			
Negative	38(15.0)	ND	
Positive	216(85.0)	ND	
Anti-HCV			
Negative	111(86.0)	ND	
Positive	18(14.0)	ND	

ND, not known.

Supplementary Table S3, HCC patient clinical features and univariate analysis of overall survival

Factors	All HCC n(%)	Death, n	MST[a] (M)	*P-value*
Grade	n=130	n=43		
High	31(23.8%)	5(11.6%)	50.7[b]	0.170
Moderate	73(56.2%)	28(65.1%)	48.000	
Low	26(20.0%)	10(23.3%)	45.000	
Smoking	n=205	n=71		
Never Smoker	172(83.9%)	60(84.5%)	69.000	0.380
Ever Smoker	33(16.1%)	11(15.4%)	98.9[b]	
Drinking	n=205	n=71		
Nondrinker	186(90.7%)	66(93.0%)	90.000	0.383
Drinker	19(9.3%)	5(7.0%)	87.0[b]	
Family history	n=309	n=123		
No	252(81.6%)	106(86.2%)	51.000	0.123
Yes	57(18.4%)	17(13.8%)	95.6[b]	
HBV	n=168	n=58		
No	30(17.9%)	11(19.0%)	27.000	0.102
Yes	138(82.1%)	47(81.0%)	86.5[b]	
HCV	n=118	n=51		
No	102(86.4%)	44(86.3%)	28.000	0.984
Yes	16(13.6%)	7(13.7%)	27.000	

[a], MST, median survival time (months). [b], mean survival time was provided when MST could not be calculated.

Supplementary Figure S1. The LD figure of the *H19* genetic polymorphisms

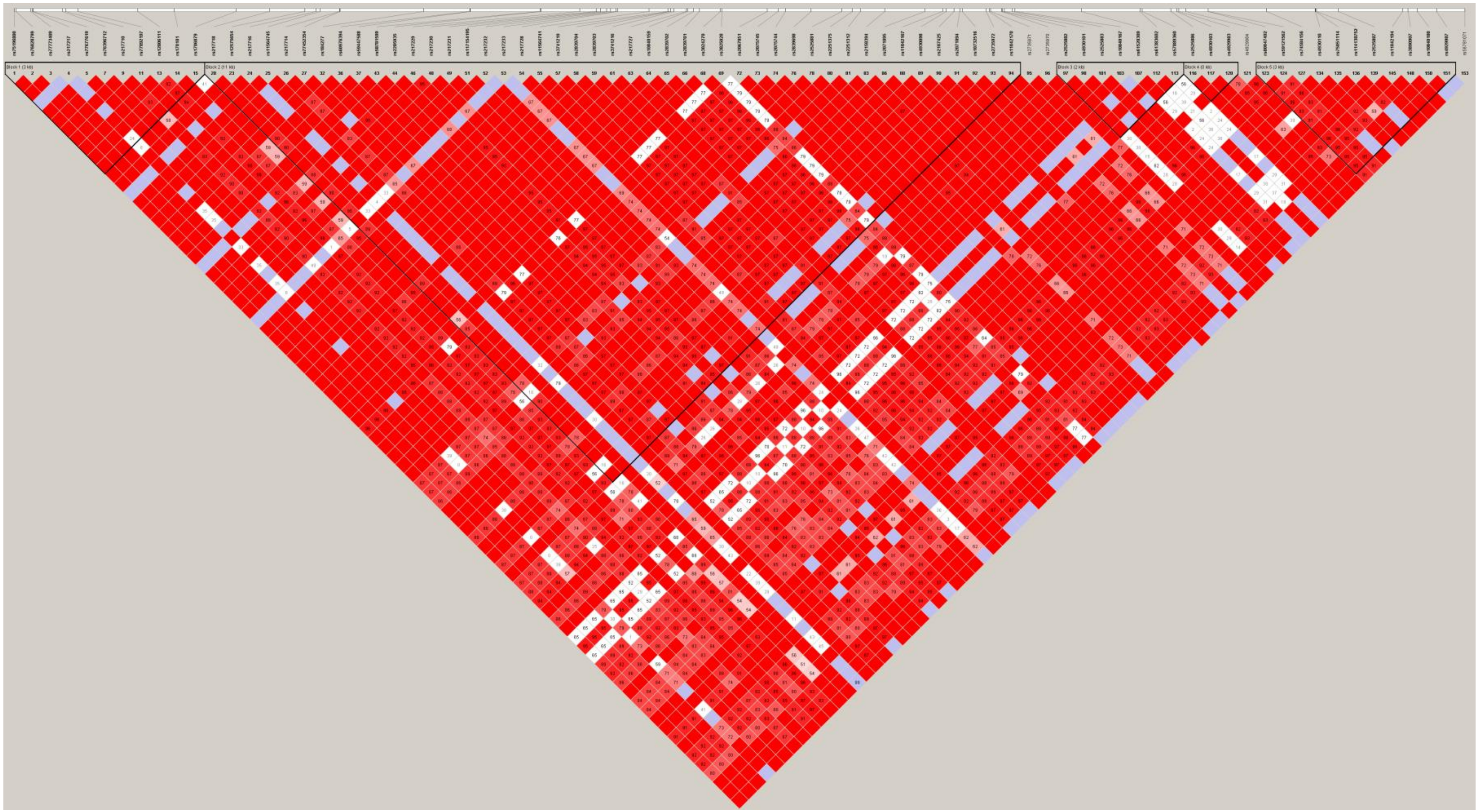

Figure legend. The studied polymorphisms of lncRNA-*H19* were selected the HapMap data. TagSNPs were selected by Tagger via Haploview with the following criteria: pairwise tagging of HapMap population with $r^2 \geq 0.8$; a minor allele frequency (MAF) ≥5%; and Chinese Han Beijing (CHB) ethnicity. And we expanded 10kbp both upstream and downstream of *H19*. Then, the LD figure of the *H19* genetic polymorphisms was shown.

Supplementary Material. The results of the haploview software for the selection of the *H19* genetic polymorphisms.

captured 81 of 81 alleles at r^^{2} >= 0.8
captured 100 percent of alleles with mean r^^{2} of 0.956
using 17 Tag SNPs in 17 tests.

Allele	Best Test	r^^{2} w/test
rs75196800	rs75196800	1.0
rs76028799	rs75196800	1.0
rs77773409	rs75196800	1.0
rs217217	rs217217	1.0
rs77677619	rs75196800	1.0
rs76396712	rs75196800	1.0
rs217710	rs217710	1.0
rs77092197	rs75196800	1.0
rs12806111	rs2525883	0.878
rs170101	rs4930101	0.858
rs1706879	rs1706879	1.0
rs217718	rs217718	1.0
rs12575654	rs2525883	0.9
rs217716	rs217716	1.0
rs11564745	rs2525883	0.9
rs217714	rs4930101	0.858
rs77452354	rs75196800	1.0
rs184277	rs4930101	0.925
rs60976394	rs217231	1.0
rs59447588	rs217231	1.0
rs58781599	rs75196800	0.962
rs2285935	rs4930101	0.9
rs217229	rs217231	0.981
rs217230	rs4930101	0.925
rs217231	rs217231	1.0
rs117154195	rs75196800	0.962
rs217232	rs217231	1.0
rs217233	rs217233	1.0
rs217728	rs2525883	0.939

rs11564741	rs75196800	0.962
rs3741219	rs4930101	0.925
rs2839704	rs4930101	0.925
rs2839703	rs4930101	0.925
rs3741216	rs75196800	0.962
rs217727	rs2525883	0.939
rs10840159	rs4930101	0.925
rs2839702	rs4930101	0.925
rs2839701	rs4930101	0.925
rs3024270	rs217710	0.864
rs3825028	rs75196800	0.89
rs2067051	rs4930101	0.95
rs2075745	rs4930101	1.0
rs2075744	rs4930101	1.0
rs2839698	rs4930101	1.0
rs2525881	rs4930101	1.0
rs2251375	rs2525883	0.8
rs2251312	rs2735971	1.0
rs2158394	rs2525883	0.979
rs2071095	rs4930101	1.0
rs11042167	rs4930101	1.0
rs4930098	rs4930101	1.0
rs2107425	rs2525883	0.816
rs2071094	rs4930101	1.0
rs10732516	rs4930101	1.0
rs2735972	rs2735971	1.0
rs11042170	rs4930101	1.0
rs2735971	rs2735971	1.0
rs2735970	rs2525883	0.816
rs2525882	rs2525882	1.0
rs4930101	rs4930101	1.0
rs2525883	rs2525883	1.0
rs10840167	rs4930101	1.0
rs61520309	rs57889360	1.0
rs61383602	rs61383602	1.0
rs57889360	rs57889360	1.0
rs2525886	rs2525886	1.0

rs4930103	rs2525887	1.0
rs4929983	rs2525887	0.852
rs4929984	rs4930101	0.823
rs80047492	rs2525887	0.961
rs59121562	rs2525887	0.961
rs74584156	rs75196800	0.888
rs4930110	rs4930101	1.0
rs75051114	rs75051114	1.0
rs114138752	rs75196800	0.888
rs2525887	rs2525887	1.0
rs11042194	rs2525887	0.961
rs3890907	rs2525887	0.801
rs10840180	rs4930101	0.95
rs4929987	rs4930101	0.95
rs56781071	rs75196800	0.852

Test Alleles Captured

<u>rs2839698:</u>

rs2067051,rs4930098,rs2075744,rs2071095,rs170101,rs184277,rs2839702,rs2525881,rs4930101,rs10840167,rs2839704,rs4929984, ,rs10840180,rs3741219,rs10840159,rs4929987,rs2285935,rs2839701,rs11042167,rs2071094,rs2839703,rs2075745,rs10732516,rs4930110,rs217230,rs217714,rs11042170

rs75196800:

rs77452354,rs75196800,rs77773409,rs117154195,rs76396712,rs114138752,rs77092197,rs58781599,rs74584156,rs11564741,rs56781071,rs3741216,rs77677619,rs3825028,rs76028799

rs2525883:

rs11564745,rs2107425,rs2251375,rs12575654,rs217727,rs217728,rs2735970,rs2525883,rs12806111,rs2158394

rs2525887:	rs11042194,rs59121562,rs2525887,rs3890907,rs4930103,rs80047492,rs4929983
rs217231:	rs217231,rs59447588,rs217232,rs217229,rs60976394
**<u>rs2735971:</u>**	rs2251312,rs2735972,rs2735971
**<u>rs3024270</u>**:	rs217710,rs3024270
rs57889360:	rs57889360,rs61520309
rs2525882	rs2525882
rs217716	rs217716
rs2525886	rs2525886
rs217233	rs217233
rs217217	rs217217

rs1706879	rs1706879
rs217718	rs217718
rs75051114	rs75051114
rs61383602	rs61383602

Then, we referred a published literature and took the intersection as the considering promising aiming SNPs. Ultimately, there were 3 SNPs covering lncRNA-H19 gene selected to proceed our study which were rs2839698 (G>A), rs2839698 (C>T), rs3024270 (G>C).
